# Civilian-military malaria outbreak response in Thailand: an example of multi-stakeholder engagement for malaria elimination

**DOI:** 10.1186/s12936-021-03995-6

**Published:** 2021-12-07

**Authors:** Michelle E. Roh, Kanyarat Lausatianragit, Nithinart Chaitaveep, Krisada Jongsakul, Prayuth Sudathip, Chatree Raseebut, Sutchana Tabprasit, Prasert Nonkaew, Michele Spring, Montri Arsanok, Parat Boonyarangka, Sabaithip Sriwichai, Piyaporn Sai-ngam, Chaiyaporn Chaisatit, Peerapol Pokpong, Preecha Prempree, Sara Rossi, Mitra Feldman, Mariusz Wojnarski, Adam Bennett, Roly Gosling, Danai Jearakul, Wanchai Lausatianragit, Philip L. Smith, Nicholas J. Martin, Andrew A. Lover, Mark M. Fukuda

**Affiliations:** 1 Malaria Elimination Initiative, Institute of Global Health Sciences, University of California, San Francisco, San Francisco, California USA; 2 Sisaket Provincial Health Office, Sisaket, Sisaket Province Thailand; 3 Royal Thai Army, Armed Forces Research Institute of Medical Sciences (AFRIMS), Bangkok, Thailand; 4 US Army Medical Directorate, Armed Forces Research Institute of Medical Sciences, Bangkok, Thailand; 5 Division of Vector Borne Disease, Department of Disease Control, Ministry of Public Health, Nonthaburi, Thailand; 6 Office of Disease Prevention and Control 10, Ministry of Public Health, Ubon Ratchathani Province, Thailand; 7 Department of Biostatistics and Epidemiology, School of Public Health and Health Sciences, University of Massachusetts-Amherst, Amherst, Massachusetts USA

**Keywords:** Military, Civilian, Malaria, Malaria outbreak investigation, Thailand, Malaria elimination, Civilian-military cooperation, Southeast Asia, Greater Mekong Subregion

## Abstract

**Background:**

In April 2017, the Thai Ministry of Public Health (MoPH) was alerted to a potential malaria outbreak among civilians and military personnel in Sisaket Province, a highly forested area bordering Cambodia. The objective of this study was to present findings from the joint civilian-military outbreak response.

**Methods:**

A mixed-methods approach was used to assess risk factors among cases reported during the 2017 Sisaket malaria outbreak. Routine malaria surveillance data from January 2013 to March 2018 obtained from public and military medical reporting systems and key informant interviews (KIIs) (n = 72) were used to develop hypotheses about potential factors contributing to the outbreak. Joint civilian-military response activities included entomological surveys, mass screen and treat (MSAT) and vector control campaigns, and scale-up of the “1–3–7” reactive case detection approach among civilians alongside a pilot “1–3–7” study conducted by the Royal Thai Army (RTA).

**Results:**

Between May–July 2017, the monthly number of MoPH-reported cases surpassed the epidemic threshold. Outbreak cases detected through the MoPH mainly consisted of Thai males (87%), working as rubber tappers (62%) or military/border police (15%), and *Plasmodium vivax* infections (73%). Compared to cases from the previous year (May–July 2016), outbreak cases were more likely to be rubber tappers (OR = 14.89 [95% CI: 5.79–38.29]; p < 0.001) and infected with *P. vivax* (OR=2.32 [1.27–4.22]; p = 0.006). Themes from KIIs were congruent with findings from routine surveillance data. Though limited risk factor information was available from military cases, findings from RTA’s “1–3–7” study indicated transmission was likely occurring outside military bases. Data from entomological surveys and MSAT campaigns support this hypothesis, as vectors were mostly exophagic and parasite prevalence from MSAT campaigns was very low (range: 0-0.7% by PCR/microscopy).

**Conclusions:**

In 2017, an outbreak of mainly *P. vivax* occurred in Sisaket Province, affecting mainly military and rubber tappers. Vector control use was limited to the home/military barracks, indicating that additional interventions were needed during high-risk forest travel periods. Importantly, this outbreak catalyzed joint civilian-military collaborations and integration of the RTA into the national malaria elimination strategy (NMES). The Sisaket outbreak response serves as an example of how civilian and military public health systems can collaborate to advance national malaria elimination goals in Southeast Asia and beyond.

## Background

In the Greater Mekong Subregion (GMS), high concentrations of mobile and migrant populations (MMP) transit and seek employment near international borders. Borders in the GMS are particular ‘hot spots’ for malaria transmission given they commonly pass through remote and densely forested areas which make them a suitable ecosystem for *Anopheles* mosquitoes. Intense cross-border population movement also increases the susceptibility of these areas to malaria outbreaks [1, 2] and multidrug-resistant *Plasmodium falciparum* parasites [3–6], affecting both civilian (e.g., rubber tappers, loggers, and miners) and military populations.

**Fig. 1:**
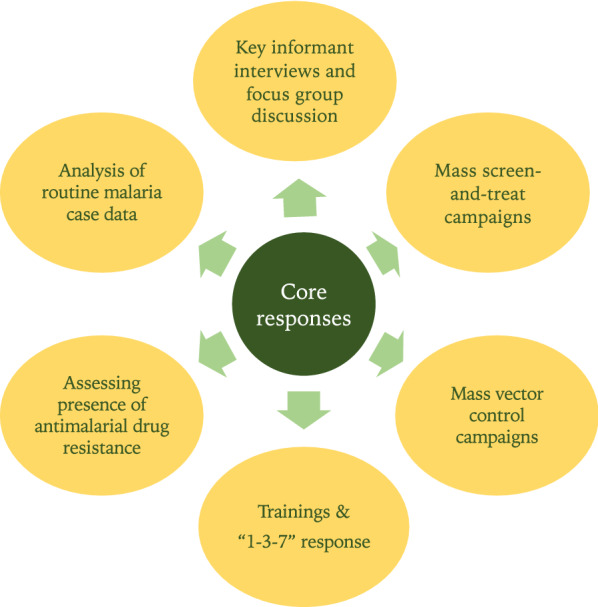
Key response activities by Emergency Operations Center during 2017–2018 Sisaket Outbreak

In April 2017, the Thai Ministry of Public Health (MoPH) was notified of an increase in malaria cases in Sisaket Province, located along the Thai-Cambodian border and an area with a significant presence of MMPs and Royal Thai Army (RTA) personnel. By May, the number of monthly reported cases surpassed the predefined epidemic threshold (two standard deviations above the mean number of monthly cases averaged across the prior four years). The Thai health authorities activated an Emergency Operations Center (EOC) to investigate the causes of the outbreak and coordinate response measures. This EOC included representatives from the MoPH’s central Division of Vector Borne Diseases (DVBD) [formerly the Bureau of Vector Borne Diseases (BVBD)]; the Sisaket Provincial Health Office (PHO) and district health staff; the RTA and US components of the Armed Forces Research Institute of Medical Sciences (RTA-AFRIMS and USAMD-AFRIMS, respectively); the Malaria Elimination Initiative at the University of California, San Francisco; and local administrative staff. The outbreak investigation and response focused on six key activities (Fig. 1) aligned with Thailand’s National Malaria Elimination Strategy (NMES) for 2017–2026 and the National Malaria Elimination Operational Plan (NMEOP) for 2017–2021 [7]. Activities included:

- Analysis of routine malaria surveillance data collected by the MoPH and RTA.
- Key informant interviews (KIIs) among malaria stakeholders.
- Molecular genotyping of antimalarial drug resistance markers (as Thai-Cambodian border is known to harbor multidrug-resistant *P. falciparum* parasites [4]).
- Scale-up of “1–3–7” reactive case detection (RACD).
- Mass screen and treat (MSAT) campaigns.
- Mass distribution campaigns of insecticide-treated bed nets (ITNs), insecticide-treated hammock nets (ITHNs), and indoor residual spraying (IRS)..

The overall aim of this study was to describe the outbreak and the key activities that followed, including collaborative activities between the public and military health sectors.

## Methods

### Study site

**Fig. 2:**
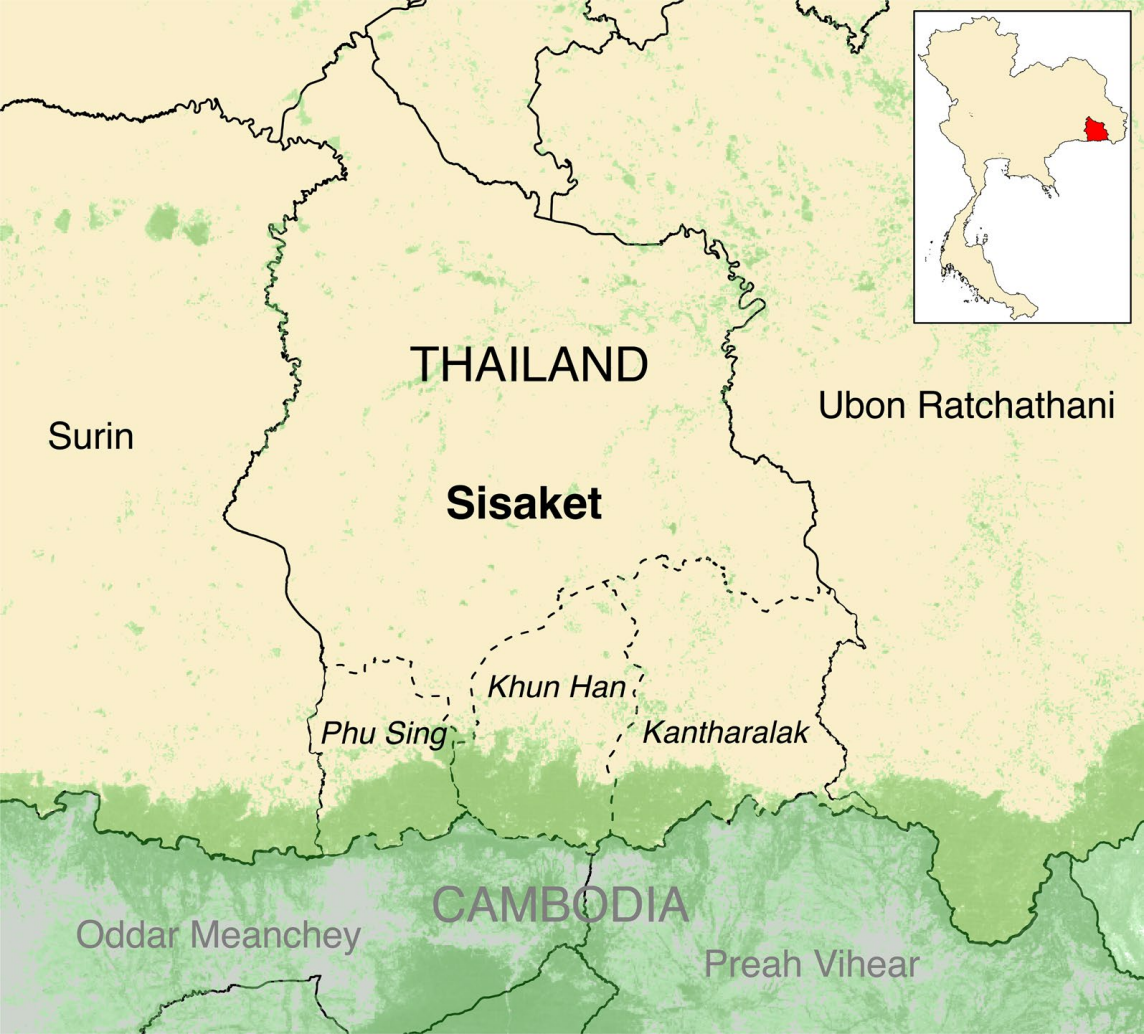
Map of Sisaket Province, Thailand. Green shaded areas indicate forested areas. Districts with the highest malaria burden (Kantharalak, Khun Han, and Phu Sing) are italicized

The investigation was conducted in Sisaket Province located in northeastern Thailand along the heavily forested Thai-Cambodian border (Fig. 2). In 2016–2017, the year prior to the outbreak, 952 malaria cases were reported in Sisaket through the MoPH’s malaria surveillance system, an estimated malaria incidence of 0.58 cases per 1000 person-years [8]. In Sisaket, cases mainly occur in the southern districts, along the Thai-Cambodian border. In these areas, malaria is highly seasonal, with two distinct bi-annual peaks: May–August and October-January and transmission is thought to be driven by forest-based exposures and MMPs [9].

## Malaria service delivery in military versus civilian health systems

Despite facing similar occupational and geographic risk factors, malaria services for civilians and military personnel are delivered through separate health systems. For civilians, malaria services are generally managed at public health facilities under MoPH. At MoPH facilities, suspected cases of malaria are confirmed either by rapid diagnostic test (RDT) or blood smear microscopy and reported directly into MoPH’s web-based malaria surveillance database, the Malaria Information System (MIS) [8]. For first-line treatment of *P. falciparum* malaria, national guidelines at the time of the outbreak recommended a three-day regimen of dihydroartemisinin-piperaquine (DHA-PPQ) and single-low dose primaquine. The first-line treatment for *Plasmodium vivax* infections was and remains a three-day regimen of chloroquine and a 14-day regimen of primaquine (PQ). Prior glucose-6-phosphate dehydrogenase testing is recommended for administration of PQ for radical cure in Thailand [10].

To identify additional asymptomatic cases and prevent onward transmission, Thailand’s NMES for 2017–2026 recommends the “1–3–7” RACD approach [11], a strategy first adopted by the national malaria elimination programme in China [12]. Under “1–3–7”, confirmed and suspected malaria cases are rapidly reported to the MIS within one day; investigation is completed within three days; and response activities are conducted within seven days. In addition to “1–3–7”, the Sisaket PHO conduct routine entomological and foci surveillance; proactive case detection; and ITN/ITHN campaigns.

For RTA personnel, malaria services are managed through the military health care system which spans a continuum of large tertiary care hospitals (generally located in urban centers) to military versions of MoPH “malaria posts”. These posts are near areas of high transmission risk for military personnel, whose occupational risk factors are parallel to MMPs. In the RTA malaria posts, suspected cases are diagnosed by RDT, confirmed by microscopy by MoPH where feasible, and treated following national treatment guidelines. Cases are reported to a passive surveillance system managed by the RTA Medical Department and monthly aggregated case counts are shared with MoPH. Occasionally, military personnel are directly diagnosed and treated at MoPH facilities, and occupational status is captured as ‘military/border police’ in the MoPH case investigation form. Military health staff engage in routine malaria control efforts, including semi-annual IRS of barracks, routine distribution of ITNs, and proactive case detection. Soldiers are advised to use topical repellents while in the field. Prior to this outbreak, “1–3–7” activities were not conducted as part of the military’s routine malaria prevention and response strategy.

## Malaria surveillance data

Routine malaria surveillance data from January 2013 to March 2018 were obtained from Thailand’s case-based MIS surveillance system [8] and RTA’s passive malaria surveillance system. Characteristics of MIS-reported cases were summarized using frequencies and column percentages for categorical variables. To generate hypotheses of the potential causes of the outbreak and to assess for any demographic shifts from the previous year, characteristics were compared between outbreak cases and cases detected in the previous year (May–July 2016). Bivariate analyses comparing characteristics between these two groups were conducted using logistic regression models and p-values <0.05 were considered statistically significant. Statistical analyses were performed using R (version 3.5.3; http://www.r-project.org/).

Passive malaria surveillance data from RTA was used to generate epidemiological curves, but risk factor analyses could not be conducted among RTA cases, as neither individual-level nor demographic data were available.

### Entomological surveys

Routine entomological surveillance data were obtained from MoPH’s Vector Borne Disease Center (VBDC) to describe the prevalence of vector species in Sisaket Province. Indoor and outdoor human landing catches were carried out in Phu Sing, Khun Han, and Kantharalak Districts.

### Key informant interviews

Key informant interviews were conducted by public health professionals from the DVBD and Sisaket PHO. Interviews were conducted in Thai by members of the study staff using a semi-structured questionnaire, with responses being captured manually by multiple observers simultaneously. Analyses of these data were conducted using TAMS Analyzer [13].

## Results

### Analysis of malaria surveillance data

#### MoPH surveillance system

**Fig. 3:**
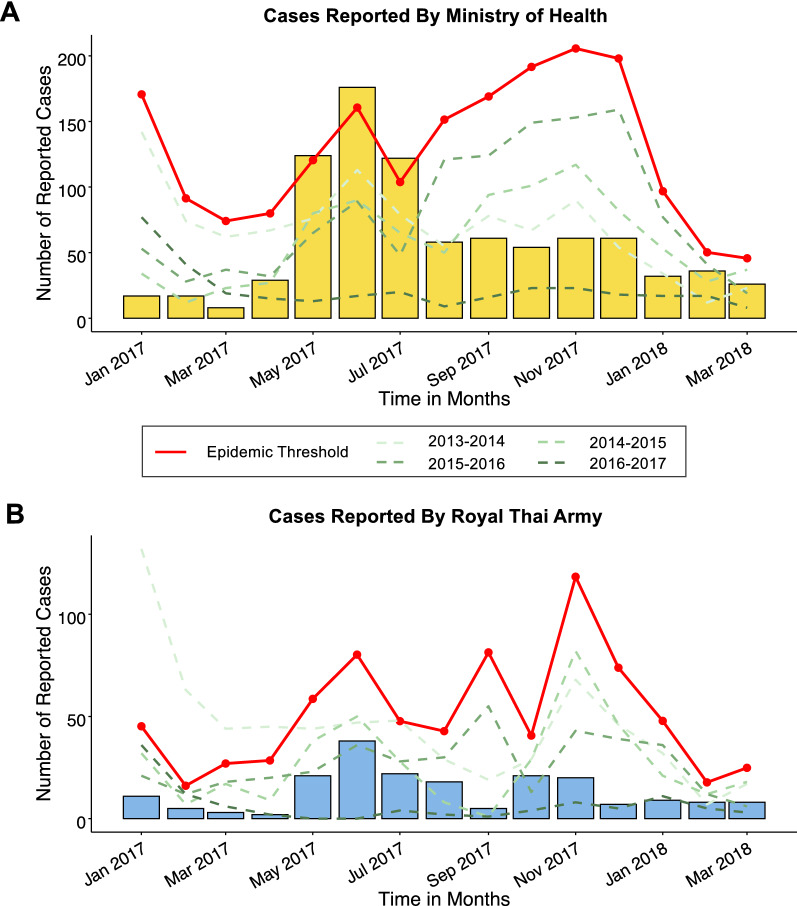
Monthly malaria cases reported by Ministry of Public Health (**A**) and Royal Thai Army (**B**). In **A**, the orange shaded bars indicate the monthly number of *Plasmodium vivax* infections and the yellow shaded bars indicate the monthly number of *P. falciparum* infections and three mixed infections reported between January 2017 and March 2018). In **B**, the blue shaded bars indicate the monthly number of monthly malaria cases, as data on species types of RTA cases was unavailable from the routine malaria surveillance data. For both **A** and **B**, the red solid line indicates the epidemic threshold which was calculated as two standard deviations above the mean number of monthly cases averaged across the prior four years. The dashed green lines indicate the number of monthly malaria cases which were reported in the prior four years

Between May and July of 2017, the monthly number of cases surpassed the epidemic threshold (two standard deviations above the mean number of monthly cases averaged across the previous four years). During this period 422 cases were reported to the MIS, a 2.2-fold increase from the previous four-year average (n = 189) and an 8.4-fold increase compared to the previous year (n = 50) (Table 1). By August 2017, monthly caseloads returned to pre-outbreak levels (Fig. 3A). The majority of outbreak cases had *P. vivax* mono-infections (73%). 95% of cases came from three districts: Khun Han (45%), Phu Sing (28%), and Kantharalak (22%), all of which lie on the mountainous and forested Thai-Cambodian border (Fig. 2). Outbreak cases were largely comprised of Thai males (87%) of working age (91% were between 15 and 59 years of age), who reported traveling outside their home village or outside of Thailand in the past two weeks (68% and 29%, respectively). Rubber tapping and military/border police were the most commonly reported occupations among cases (62% and 15%, respectively).

**Table 1:** Bivariate analyses of risk factors associated with outbreak cases (May–July 2017) compared to cases reported in the previous year (May–July 2016)

Characteristics	Outbreak cases (n = 422)	Cases from the previous year (n = 50)	OR [95% CI]	p-value
Species				
*Plasmodium vivax*	308 (73)	27 (54)	2.32 [1.27, 4.22]	0.006
*Plasmodium falciparum*	113 (27)	23 (46)	Ref	
Mixed	1 (0.002)	0 (0)	–^a^	< 0.001
District of residence, n (%) ^b^				
Kantharalak	92 (22)	10 (20)	1.12 [0.54, 2.32]	0.77
Khun Han	191 (45)	32 (64)	0.47 [0.25, 0.85]	0.014
Phu Sing	116 (28)	5 (10)	3.41 [1.32, 8.81]	0.011
Other	23 (5)	3 (6)	0.90 [0.26, 3.12]	0.87
Nationality, n (%)				
Thai	412 (98)	49 (98)	Ref	
Cambodian	10 (2)	1 (2)	1.19 [0.15, 9.50]	0.87
Gender, n (%)				
Male	368 (87)	48 (96)	Ref	
Female	54 (13)	2 (4)	3.52 [0.83, 14.91]	0.087
Age in years, n (%) ^b^				
0–14	18 (4)	0 (0)	--^a^	--
15–59	386 (91)	48 (96)	0.45 [0.10, 1.91]	0.28
≥ 60	18 (4)	2 (4)	1.07 [0.24, 4.75]	0.93
Travel history ^b^				
No travel	15 (4)	8 (16)	0.19 [0.08, 0.48]	< 0.001
Within Thailand	285 (68)	33 (66)	1.07 [0.58, 1.99]	0.83
Outside Thailand	122 (29)	9 (18)	1.85 [0.87, 3.93]	0.11
Main occupation ^b^				
Rubber tapper	263 (62)	5 (10)	14.89 [5.79, 38.29]	< 0.001
Soldier/police	62 (15)	11 (22)	0.61 [0.30, 1.26]	0.18
Child/student	25 (6)	2 (4)	1.51 [0.35, 6.58]	0.58
Rice farmer	15 (4)	23 (46)	0.04 [0.02, 0.09]	< 0.001
Other	57 (14)	9 (18)	0.71 [0.33, 1.54]	0.39

Data were extracted from the Thai Ministry of Public Health’s Malaria Information System (MIS). Outbreak cases were defined as cases reported during the months that surpassed the epidemic threshold

*OR* odds ratio

^a^Odds ratios were not estimated due to zeroes in cell counts

^b^Dummy variables were used for characteristics with more than two categories. For example, the comparison (reference) group for rubber tappers was non-rubber tappers

Of the 422 cases captured through the MIS during the outbreak period, 280 (66%) cases had data available on travel history and use of personal protective equipment. All but one case (n = 279) reported spending at least one night outside of their home in the previous two weeks, with most cases reporting sleeping in a hut outside their village (n = 193; 69%) or in the forest (n = 82; 29%). Commonly reported reasons for travel included: rubber tapping (n = 141; 50%); military operations/border patrol (n = 52; 19%); forest foraging (n = 38; 14%); hunting (n = 24; 9%); or pilgrimage/meditation retreat (n = 17; 6%). Overall, 60% percent of cases reported sleeping under an ITN (n = 161), though ITN use significantly differed by overnight dwelling (p < 0.001), such that reported ITN use was high among cases that reported sleeping in a hut outside their village (77%), but low among those that reported sleeping in the forest (24%).

To assess any demographic shifts or changes in risk behaviors that may have contributed to this outbreak, characteristics of outbreak cases were compared with cases from the previous year (May–July 2016; n = 50) (Table 1). Compared to the previous year, outbreak cases had higher odds of being infected with *P. vivax* (OR=2.32 [95% CI=1.27, 4.22]; p = 0.006) and report rubber tapping as their main occupation (OR= 14.89 [95% CI: 5.79–38.29]; p < 0.0001), but lower odds of not traveling in the previous two weeks (OR = 0.19 [95% CI: 0.08–0.48]; p < 0.001) and being rice farmers (OR=0.04 [0.02, 0.09]; p < 0.001). The proportion of military and border police cases did not statistically significantly differ between years (15% versus 22%; p = 0.18).

#### RTA surveillance system

Aggregate case counts from January 2017 to March 2018 were collected from RTA’s passive malaria surveillance system. Similar to MoPH cases, RTA observed a substantial increase of cases during the outbreak period compared to the previous year (n = 4; a 20.3-fold increase) (Fig. 3B). However, the number of cases during the outbreak period were much lower compared to the previous four-year average (n = 187; 0.4-fold decrease). Similar to the MoPH epidemiological curve, RTA cases peaked between May and July, though a second, smaller peak was observed between October and November, which was not found in MoPH trends (Fig. 3A).

### Entomological surveys

In November 2017, a few months after the peak outbreak period, MoPH staff conducted standardized entomological surveys in nine villages within Phu Sing, Khun Han, and Kantharalak districts. *Anopheles barbirostris*, *Anopheles philippinesis*, and *Anopheles maculatus* (which are generally exophagic vectors with varied peak biting hours [14–18]) were the primary vector species across sites. Although insecticide-susceptibility data on local populations in Sisaket was not available at the time of the outbreak investigation, entomological data from a neighboring province (Ubon Ratchathani), found these vectors to be highly susceptible to both deltamethrin and permethrin, insecticides of the pyrethroid family commonly used in ITNs and IRS [19].

### Key informant interviews

In December 2017, the DVBD and Sisaket PHO conducted key informant interviews to contextualize and formulate hypotheses of the potential contributors to the outbreak (Table 2). Interviews were conducted in Kantharalak, Phu Sing, and Khun Han districts and consisted of one group interview and 71 rapid, semi-structured interviews among MoPH health staff (n = 45), military health staff (n = 1), recent malaria patients (n = 15), and village leaders (n = 10). Themes of interviews included: forest-based travel and occupations, general care-seeking behavior, and any observed changes in weather or occupational factors.

**Table 2:** Illustrative responses from key informant interviews and focus groups

Query	Response
What types of higher-risk activities have you been involved with during the last month?	“Many different things: forest fringe farming; sleeping in rice field/farm huts; hunting; fishing; and mushroom collecting.” (Phu Sing; male, recent malaria patient). “Forest fringe farming; sleeping in rice field/farm huts.” (Kantharalak; female, recent malaria patient).
What types of activities do you think led to the recent changes in malaria cases here?	“Protection measures are not good as there is no bed net use during rubber tapping, and mosquitoes bite during rubber tapping.” (Phu Sing; male, village leader.) “Parents bring their children to work in rubber plantation farm more and patients didn’t protect themselves.” (Phu Sing; female, village leader).
What increases risk in the high-risk populations?	“It’s their occupation--work in the forest, rubber tapping stay overnight in the rubber plantation farm, with increased mosquitoes. We need a campaign for people not to stay overnight in the forest.” (Phu Sing, male village leader). “Work in the forest hunting, foraging, or stay overnight in the forest in the cave. People can’t use repellents as animals will know. Soldiers can use hammocks, but can’t use bed nets since they’re white colour, very dangerous as they can be seen from far away.” (Khun Han; male health staff).

The general consensus among interviewees was that the epidemic was driven by the recent maturation of trees on local rubber plantations. It was reported that many rubber trees in the area were planted approximately ten years ago and were now mature enough to produce sufficient amounts of latex for harvesting. Health staff and recent malaria patients also suspected illegal rosewood logging operations and other forest-based activities were contributors to malaria transmission, but no further information was available on the magnitude or impact of these activities.

Overall, the district health staff appeared to have the richest knowledge about recent changes in malaria transmission. This included mentioning that few of the cases were appearing outside the usual demographics, including the mention of fatalities by several key informants, but nearly all district staff stated that rubber plantation workers clearly constituted the majority of outbreak cases. Moreover, families that work and live on rubber plantations themselves were specifically highlighted by several interviewees. Major risk factors identified were “staying in the huts at rubber plantations”; “living in the forest and rubber tree farm and not taking enough care from mosquito bites”; and anyone “living in the jungle at nighttime”.

While the self-reported use of topical repellents and ITNs was frequent among cases, few interviewees reported the use of ITHNs or long-sleeved clothing while in the forest. The reasons provided for not using these vector control interventions included: difficulty in using interventions in the forest; being too busy working; or simply forgetting to use personal protective equipment. Topical repellent was also reported to “scare away animals while hunting,” and the one soldier reported that “when on patrol, it’s difficult to prevent [malaria].” However, only a few individuals knew that mosquito bites specifically were the cause of malaria, with “dirty water” being a common response.

The military-specific interview was limited to a single military clinic with one medic and three recent malaria patients. All three military malaria cases stated that use of ITNs and topical repellents was common. The medic interviewee stated that each military squad on patrolling duties had a medical kit that included both RDTs and artemisinin-based combination therapies and for cases diagnosed at the military clinic, all *P. falciparum* cases were referred to the closest MoPH hospital, while confirmed *P. vivax* cases received chloroquine directly and remained warded in the military hospital for the duration of their 14-day PQ regimen to ensure medication adherence.

### Drug resistance surveys

During the same period Cambodia replaced DHA-PPQ with artesunate-mefloquine as the first-line treatment for uncomplicated *P. falciparum* malaria (in 2016) [20], USAMD-AFRIMS began molecular surveillance of anti-malarial drug resistance in a military camp located in the southwestern region of Sisaket, close to the Cambodian border. These data have been reported elsewhere [21], but in summary, because of the relatively low numbers of *P. falciparum* cases recorded during outbreak, *P. falciparum* resistance was not determined to be the underlying cause of the outbreak.

### Outbreak response activities

**Fig. 4:**
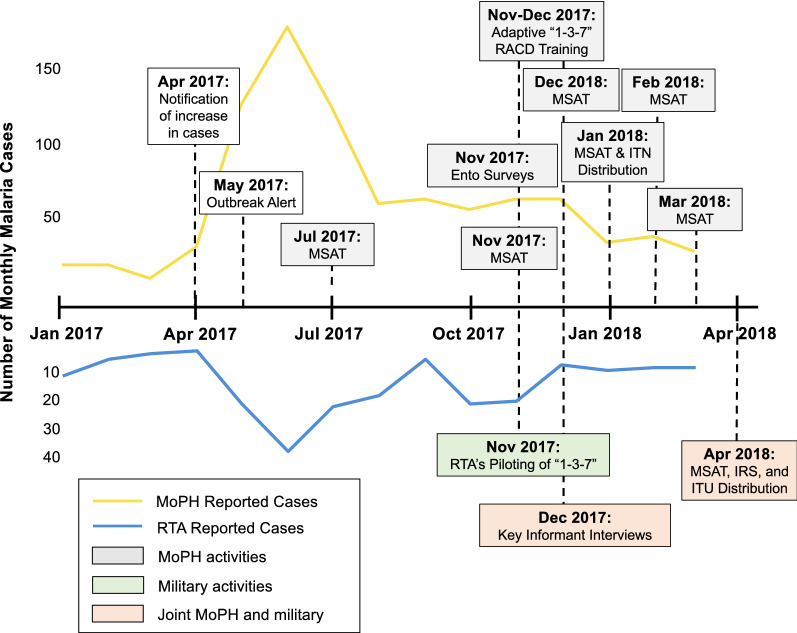
Timeline of outbreak investigation and response events. Yellow and blue lines indicate the number of monthly malaria cases reported by the Ministry of Public Health and the Royal Thai Army, respectively. Shaded boxes indicate activities conducted by MoPH (grey), military (green), or both (orange)

During the outbreak, a series of meetings were held between members of the EOC to discuss response activities and potential collaborations between public and military health sectors. Activities included MSAT campaigns, mass vector control campaigns, scale-up of adaptive “1–3–7” RACD among civilians, and implementation of “1–3–7” among military personnel (Fig. 4).

#### Mass campaigns

Between July 2017 and April 2018, MoPH and USAMD-AFRIMS conducted six rounds of mass screen and treat (MSAT) campaigns across several active foci villages (Fig. 4). During these campaigns, a total of 6,299 individuals were tested via microscopy and/or polymerase chain reaction (PCR). Parasite prevalence across rounds ranged from zero to 0.7%. In January 2018, MoPH conducted large-scale ITNs and ITHNs campaigns across 103 villages in Kantharalak, Khun Han, Phu Sing, and Khukhan districts. In April 2018, MoPH, RTA Medical Department, and USAMD-AFRIMS distributed insecticide-treated uniforms and conducted IRS and MSAT campaigns across eight military camps (Fig. 4).

#### Scale-up of “1–3–7” activities

Between November and December 2017, a series of trainings were led by the MoPH to improve case management and increase capacity for “1–3–7” response in Sisaket Province. By May 2018, Sisaket public health staff were re-trained on new MoPH guidelines that recommended a more adaptive approach to “1–3–7”. Rather than conducting RACD around index cases’ household, the adaptive approach conducted RACD among 50 other at-risk persons with a known association to the index case [22]. Indicators of completeness and timeliness of “1–3–7” after re-trainings are presented in Table 3. After trainings, modest changes were observed in the completeness and timeliness of case investigations and responses. According to public health staff, the main reasons for delays in response activities were lack of human resources at the proper levels and high caseloads associated with the outbreak.

**Table 3:** Indicators of completeness and timeliness of “1–3–7” after refresher trainings resulting from the 2017–2018 Sisaket outbreak. Data retrieved from Thailand’s Malaria Information System [8]

“1–3–7” Indicators	May 2017 to Apr 2018	May 2018 to Apr 2019	% difference between years		p-value^1^	
Total cases	1042	829	− 20%		–	
Completeness						
N (%) investigated	914 (88)	762 (92)	+4%		0.003	
N (%) requiring response^2^	473 (52)	421 (55)	+3%		0.15	
N (%) responded to^3^	224 (47)	225 (53)	+6%		0.069	
Timeliness						
Cases reported within one day	401 (38)	445 (54)	+16%		<0.0001	
Cases investigated within three days^2^	903 (99)	739 (97)	− 2%		0.009	
Responded to within seven days^4^	120 (54)	110 (49)	− 5%		0.32	

^1^ P-values computed using Chi-squared test to compare proportions between 2018–2019 and 2017–2018 cases

^2^ Denominator for indicator is the number of cases investigated

^3^ Denominator for indicator is the number of cases requiring a response

^4^ Denominator for indicator is the number of cases responded to

In November 2017, to align with national malaria elimination goals, RTA-AFRIMS began piloting the “1–3–7” approach among Sisaket-based RTA personnel. Implementation and design of the “1–3–7” approach were closely aligned with guidelines provided by the MoPH, including the adaptation of MoPH case investigation forms. Cases were reported and investigated on the same day of diagnosis and day 7 responses were allocated to the neighbouring geographic area of the index case. Response activities included reactive case detection around the index case’s base using microscopy, IRS of barracks, and providing malaria education for soldiers.

Between November 2017 and September 2018, a total 168 case reports from the RTA were investigated. Of these total cases, 150/168 (89%) were *P. vivax* infections, 3 (2%) were *P. falciparum*, and 15 (9%) were mixed species infections. Data from 166/168 investigations (98%) were available for analysis. All cases were male (100%), approximately 25 years of age (SD: 7; range: 21-58), stationed across three military bases. Vector control use was high among soldiers: self-reported ITN, topical repellent, and IRS use (in the prior six months) was 90%, 69%, and 95%, respectively. Of the 166 cases that had travel history information available, 76 (46%) reported traveling to the forest in the previous month prior to the onset of febrile illness, with 100% reporting military missions as the purpose for travel. Though all reported cases were investigated, only 10 (6%) day 7 responses were conducted. RTA outbreak personnel indicated the low day seven response rate was primarily due to inability to follow up cases who were deployed on active military duties.

## Discussion

In April 2017, the Thailand MoPH was alerted to an increase in malaria cases in Sisaket Province. Between May and July 2017, the case burden surpassed the epidemic threshold. An EOC, which comprised of both public and military health officials, was launched to investigate and respond to the outbreak. During the three-month outbreak period, public and military health sectors observed an 8.4-fold and 20.3-fold higher caseload compared to the previous year, respectively. Based on case investigation data collected through MoPH, outbreak cases were mostly Thai males of working age, infected with *P. vivax*, who engaged in forest-related activities (predominantly rubber tappers and military/border police). Relative to national targets (55% in 2018) [11], self-reported ITN use was high among civilians (60%) and military (84%). However, upon further investigation of MoPH case investigation surveillance data, ITN use was sub-optimal among travelers who slept in the forest (24%) compared to those sleeping in huts (77%). Routine entomological surveys conducted in the neighbouring province indicated the presence of mostly exophagic vector species with varied biting hours, further confirming cases were likely unprotected during their time in the forest. Based on qualitative interviews conducted after the outbreak, district-level health workers appeared to have the richest knowledge of the malaria epidemiology, which supported findings from routine surveillance data. Data from antimalarial drug resistance surveillance systems collected around the same period detected piperaquine-associated drug resistance mutations (PM2). Though this finding does not constitute definitive evidence of treatment failure nor the reason for the documented outbreak (as most cases during the outbreak were *P. vivax*), these data along with molecular genotyping results from other groups supported the Thai MoPH’s decision to change the first-line ACT for *P. falciparum* in Sisaket and neighboring Ubon Ratchathani Provinces to pyronaridine-artesunate in July 2019. This decision proved to be prescient, since clinical DHA-PPQ treatment failures were subsequently reported in the region [23].

Consistent with other settings in Thailand [24] and the GMS [25, 26], this study suggests the outbreak was driven by increased population movement of rubber tappers and military near the forest and forest fringes of the Thai-Cambodian border. Though the source of the outbreak remains unknown, the parallel epidemiological curves between MoPH and RTA indicates a strong link between these two MMPs, suggesting an outbreak in civilians will likely lead to an outbreak among military, and vice versa. Vector control use (e.g., ITNs) during high-risk period/locations was similarly low in these two groups. Such observations indicate both MoPH and RTA should consider evaluating additional malaria prevention methods to supplement current activities that target static transmission, especially ones that can accommodate the practical realities of working in forested areas. These could include the chemoprophylaxis strategies (e.g., individual chemoprevention, focal mass drug administration); spatial repellents; wearing long-sleeve shirts, trousers, and socks; insecticide-treated clothing; and/or alternative ways for high-risk populations to access prevention and treatment services (e.g., via peer navigators or mobile malaria workers that can test and treat in forest fringe areas during the high malaria season) [26, 27]. In addition, improving upon prompt case management and timeliness of “1–3–7” activities, particularly in border and forested areas, would help to predict and mitigate future outbreaks [28].

## Study limitations

This study was subject to several limitations. First, the malaria surveillance data obtained from the military health system was limited to aggregated case counts, and risk factors among military outbreak cases could not be assessed. Second, due to logistical challenges, a formal case-control study which would have allowed a more rigorous evaluation of potential risk factors, especially if risk factors differed between RTA and civilians [29]. Third, conclusions from key informant interviews may be subject to selection bias, as interviews were conducted amongst those who were willing or able to come to interview sites. Furthermore, responses from recent patients may have also been subject to social desirability bias (particularly if recent cases had participated in semi-legal or illegal activities) and recall bias, due to the delayed timing of the interviews (December) relative to the peak outbreak period (May–July). Fourth, rigorous impact evaluations of the joint outbreak response activities were outside the scope of this work and it is unclear from this study what effect these response activities had on the overall outbreak and proceeding malaria season. However, it is unlikely that MSAT campaigns had a major impact on outbreak cessation, as cases were already reducing by the time the first MSAT campaign was initiated and little to no cases were found during these campaigns. Similarly, the ITN campaign and adaptive “1–3–7” RACD approaches were also conducted after the outbreak, though it is possible that these activities may have prevented a second seasonal peak in October and January.

## Need for continued civilian-military collaboration

Despite the study limitations, an important outcome of this investigation was the establishment of public and military health sector collaboration toward outbreak mitigation. Within two months of the outbreak alert, MoPH and RTA began conducting joint response activities (e.g., MSAT and vector control campaigns) and the military began introducing elimination strategies into their regular malaria prevention and response activities. Though it is unclear whether these activities led to a reduction in case burden and possible prevention of a second seasonal peak between October and January, this is the first time in peer-reviewed literature to report the inclusion of military into national malaria outbreak response activities and the adoption of specific malaria elimination strategies by the Thai military in support of their civilian counterparts.

In the GMS, militaries represent an important, but often underrecognized reservoir of malaria transmission [30]. Because malaria services tend to be delivered through separate military and civilian systems, national malaria elimination goals and strategies have not always aligned. Facing the urgency of a mounting malaria outbreak in Sisaket, the RTA was swift in their response by sharing surveillance data with MoPH counterparts, piloting a “1–3–7” RACD approach, and participating as members of the EOC. Similar to successful cross-border collaborations [6, 31, 32], civilian-military collaborations will require substantial political commitment by both parties. In Sisaket, civilian-military collaborations were made possible through regular meetings between high-level MoPH and RTA officials, the designation of trusted local public health staff to provide technical support and resources to military health personnel, and through joint research efforts by USAMD- and RTA-AFRIMS to pilot malaria elimination research activities among the military. This case study may serve as a blueprint for other countries to proactively begin discussions between public and military malaria programmes. Outputs from these collaborations will greatly benefit both acute crisis response (as malaria outbreaks in the military are likely to affect civilian populations and vice versa) and ensure that military malaria activities are fully aligned with national malaria elimination goals and strategies. In addition to national civilian-military cooperation, better cross border collaboration will be needed to greatly accelerate progress toward national and regional malaria elimination goals, particularly in Sisaket, where transmission is likely driven in part by cross-border movement along the Thai-Cambodian border [33].

## Conclusions

In 2017, an outbreak of malaria among civilian and military persons occurred in Sisaket Province, Thailand. Joint outbreak investigations by military and public health sectors found forest-related activity (e.g., rubber tapping, border patrolling, and military operations in the forest) was a major factor in the observed increase in cases. Civilians and military personnel both reported high ITN use, though use was limited to the cases’ home or military base, suggesting alternative interventions were needed during the period they engaged in forest-related activities. Though monthly caseloads returned to pre-outbreak levels by August 2017, the outbreak resulted in strong “civ-mil” collaborations, better integration of the military into national malaria elimination goals and strategies, and a practical opportunity to implement Thailand’s National Malaria Elimination Operational Plan (NMEOP). Collaborative “civ-mil” efforts as observed during this outbreak can serve as an initial model for other countries in the GMS and Southeast Asia that aim to eliminate malaria by 2030.

## Data Availability

The data generated and analysed in the current study may be available from the corresponding author upon reasonable request and with permissions from the Thai MoPH and/or RTA.

## Abbreviations

ACT: Artemisinin-based combination therapy
AFRIMS: Armed Forces Research Institute of Medical Sciences
DHA-PPQ: Dihydroartemisinin-piperaquine
DVBD: Division of Vector Borne Diseases, MoPH Thailand
EOC: Emergency Operations Center
GMS: Greater Mekong Subregion
IRS: Indoor residual spraying
ITHN: Insecticide-treated hammock net
ITN: Insecticide-treated net
KII: Key informant interviews
MoPH: Ministry of Public Health, Thailand
MIS: Malaria Information System
MMP: Mobile and migrant populations
MSAT: Mass screen and treat
NMEOP: National Malaria Elimination Operational Plan
NMES: National Malaria Elimination Strategy
PCR: Polymerase chain reaction
PHO: Provincial Health Office
PM2: Plasmepsin copy number 2
PQ: Primaquine
RACD: Reactive case detection
RDT: Rapid diagnostic test
RTA: Royal Thai Army
USAMD: United States Army Medical Directorate
VBDC: Vector Borne Disease Center
